# Crossing fitness valleys: empirical estimation of a fitness landscape associated with polymorphic mimicry

**DOI:** 10.1098/rspb.2016.0391

**Published:** 2016-04-27

**Authors:** Mónica Arias, Yann le Poul, Mathieu Chouteau, Romain Boisseau, Neil Rosser, Marc Théry, Violaine Llaurens

**Affiliations:** 1 Institut Systématique, Evolution, Biodiversité, UMR 7205 MNHN-CNRS-EPHE-UPMC- Sorbonne universités, Muséum National d'Histoire Naturelle, Bâtiment d'entomologie, CP050, 57, rue Cuvier, 75005 Paris, France; 2 UMR CNRS 7179, CNRS-MNHN MECADEV, Muséum National d'Histoire Naturelle, 1, avenue du petit château, 91800 Brunoy, France; 3 Département de Biologie, Ecole Normale supérieure, 75 005 Paris, France; 4 Department of Biology, University of York, Wentworth Way, York YO10 5DD, UK

**Keywords:** passion-vine butterfly, aposematism, dominance, linkage disequilibrium, generalization, heterozygote

## Abstract

Characterizing fitness landscapes associated with polymorphic adaptive traits enables investigation of mechanisms allowing transitions between fitness peaks. Here, we explore how natural selection can promote genetic mechanisms preventing heterozygous phenotypes from falling into non-adaptive valleys. Polymorphic mimicry is an ideal system to investigate such fitness landscapes, because the direction of selection acting on complex mimetic colour patterns can be predicted by the local mimetic community composition. Using more than 5000 artificial butterflies displaying colour patterns exhibited by the polymorphic Müllerian mimic *Heliconius numata*, we directly tested the role of wild predators in shaping fitness landscapes. We compared predation rates on mimetic phenotypes (homozygotes at the supergene controlling colour pattern), intermediate phenotypes (heterozygotes), exotic morphs (absent from the local community) and palatable cryptic phenotypes. Exotic morphs were significantly more attacked than local morphs, highlighting predators' discriminatory capacities. Overall, intermediates were attacked twice as much as local homozygotes, suggesting the existence of deep fitness valleys promoting strict dominance and reduced recombination between supergene alleles. By including information on predators' colour perception, we also showed that protection on intermediates strongly depends on their phenotypic similarity to homozygous phenotypes and that ridges exist between similar phenotypes, which may facilitate divergence in colour patterns.

## 1. Background

The origin and persistence of adaptive polymorphisms is a puzzling question for evolutionary biologists [1–4]. Adaptive polymorphisms can be defined as several coexisting phenotypes within a population, which correspond to fitness peaks in an adaptive landscape. Such landscapes may comprise one or many dimensions, depending on the complexity of the adaptive trait. Understanding the evolution of adaptive polymorphism in a complex trait is especially challenging, because natural selection can act on different features of the trait and thus on multiple dimensions of the fitness landscape. Therefore, the exploration of new adaptive peaks in such a landscape usually requires coordinated changes in multiple axes to cross fitness valleys [5]. Gradual changes alone are thought to be generally unable to bridge such fitness valleys [6]. Mechanisms such as epistasis (i.e. when several neutral or deleterious mutations produce fitness benefits when they co-occur) [7] or large size mutation followed by gradual fine-tuning [8] are more likely to allow shifts to new adaptive peaks. Evolutionary forces such as natural selection and genetic drift [5] are also thought to allow peak shifts. For example, landscapes become less rugged when selection is relaxed, allowing populations to drift across fitness valleys and arrive at new adaptive peaks.

A number of studies of fitness landscapes in natural populations have demonstrated low fitness of intermediate phenotypes. For example, recently diverged *Antirrhinum* species exhibit diverse floral phenotypes that comprise a U-shaped adaptive ‘mountain chain’ in genotypic space, which allows flower colours to evolve along ridges while circumventing non-adaptive valleys [9]. Phenotypes co-occurring within natural populations were considered as adaptive, whereas phenotypes not found within the sampling localities were assumed to fall in fitness valleys. However, fitness itself was not actually estimated. Another fitness landscape description focused on variation in jaw shape in *Cyprindon* pupfish and the consequences for adaptive radiation. Two fitness peaks were found; a broad adaptive peak used by generalist species with an overall lower fitness height than the narrow adaptive peak used by specialist species [10]. However, despite such studies, an understanding of the evolutionary processes leading to the appearance and persistence of multiple distinct adaptive peaks within populations of the same species remains challenging. This is because random mating among the different phenotypes within a population should impede the persistence of several local adaptive peaks. Here, we combined a direct empirical estimation of fitness with a precise phenotypic description, in order to characterize how selection shapes a multi-peak fitness landscape in a polymorphic system.

Polymorphism of mimetic warning signals in unpalatable species provides an ideal system to investigate the fitness landscape associated with adaptive polymorphisms within natural populations. Unpalatable species often share similar conspicuous colour patterns that serve as a warning, thereby sharing the costs associated with predators' learning. This is known as Müllerian mimicry [11]. The protection provided by a particular colour pattern is therefore tightly linked to its local abundance, following a positive frequency-dependent selection [12]. For example, by translocating individuals [13–15] or using artificial prey in the wild [16–18], several authors have reported a fitness advantage for locally abundant warning signals over rare ‘exotic’ signals. The direction and relative strength of selection acting on colour pattern in a polymorphic mimetic species are thus closely related to the abundance of the different warning signals displayed by local mimetic communities.

The strength of selection exerted by predators depends on their capacity to recognize and avoid frequently encountered warning signals. Although comimics rarely look exactly the same [19,20], imperfect mimics may still benefit from protection either because the differences between their warning signals are imperceptible to predators [21], or because they share some particular traits that predators learn to associate with unprofitable prey [22,23]. Estimating the extent to which predators generalize these variations is therefore essential to describe the width of the fitness peaks associated with mimetic warning signals. Laboratory studies have shown that the level of generalization can vary according to several features of both predator and prey communities. If many predators are inexperienced [24] or reluctant to taste new items [25], their generalization range is expected to be larger, decreasing the strength of selection for perfect mimicry. Additionally, complex communities of toxic species displaying multiple distinct aposematic signals seem to trigger a broader generalization range than less variable communities [26,27]. The presence of few distinguishing features associated with unpalatability has been shown to be sufficient to generate predator avoidance, favouring the survival of imperfect mimics that carry such features but vary otherwise [26]. For instance, wasp-mimic hoverflies might get protection by exhibiting black and yellow stripes similar to their model, but have body shapes and flying behaviour that can reveal them as harmless [28].

Mimetic warning signals that vary in patch size, shape and colour are therefore an example of a complex trait whose variation requires precise orchestration of several developmental pathways in time and space. The genetic architecture of mimetic coloration has been extensively studied in Neotropical *Heliconius* butterflies and repeated recruitments of homologous genes have been observed throughout the genus, suggesting that a conserved genomic architecture underlies a huge diversity of mimetic colour patterns [29–31]. In most *Heliconius* species, a single mimetic colour pattern is usually fixed within populations by strong purifying selection [32]. Different components of the colour pattern in such *Heliconius* species are usually controlled by several unlinked loci (see [33] for a review). By contrast, different mimetic colour patterns are controlled by a single locus in *Heliconius numata* [34], a species that exhibits a striking polymorphism within populations [35]. This polymorphism is puzzling, because the positive frequency-dependent selection imposed by Müllerian mimicry should drive one of the colour pattern morphs to fixation [36]. An estimation of the fitness landscape associated with polymorphic mimicry is therefore needed to understand the selective pressures acting on colour pattern variations, and their effect on the underlying genetic architecture, including dominance relationships and recombination rate.

*Heliconius numata* polymorphism is thought to be driven by balancing selection on mimetic interactions. Each morph of *H. numata* is a highly accurate mimic of a coexisting species from the distantly related genus *Melinaea* [37]. The frequencies of *H. numata* morphs in each locality are correlated with the abundances of the corresponding *Melinaea* species [38], which are highly heterogeneous at small geographical scales. Each morph therefore stands on a different fitness peak in a multi-peak landscape driven by the local mimetic communities. Different wing colour pattern morphs in *H. numata* are mainly controlled by a single genomic region referred to as the supergene *P* [39], which contains *ca* 20 genes and produces several distinct haplotypes. Recombination within this supergene is limited because of chromosomal inversions [34]. Given the high number of morphs present in each locality, a high frequency of heterozygotes at the supergene *P* is found in natural populations of *H. numata*. Since heterozygotes carry two different haplotypes encoding strikingly different phenotypes, they can potentially express intermediate non-mimetic morphs. However, strict dominance among haplotypes is generally observed between sympatric alleles, producing locally mimetic heterozygous phenotypes [40]. This contrasts with the weak dominance interactions observed between haplotypes from different populations, and suggests that the strict dominance among sympatric haplotypes results from selection exerted by predators against intermediate, non-mimetic heterozygous phenotypes [40]. However, the existence of such fitness valleys among adaptive peaks corresponding to the different mimetic phenotypes remains uncharacterized: the height, slope and width of the peaks needs to be investigated to infer the strength of selection acting on different pattern elements. Using artificial butterflies, we explored the shape of the fitness landscape associated with polymorphic mimicry. We directly estimated selection by wild predators on intermediate heterozygous phenotypes (the products of rare events of recombination or incomplete dominance) which exhibited varying levels of resemblance to their corresponding homozygotes.

Using artificial prey in the wild with controlled wing colour patterns allows direct estimations of predation risk that take into account the entire complexity of actual prey and predator communities. Artificial prey items have already been successfully used to infer predation pressure in mimetic organisms including butterflies [18,41] and frogs [16]. Here, we created artificial butterflies displaying intermediate, heterozygous phenotypes and precisely quantified their resemblance to homozygotic mimetic phenotypes using a new methodology that accounts for both colour perception and pattern variation. The conspicuousness of a warning signal is tightly correlated to the brightness and colour contrast of adjacent patches [42]. To accurately quantify variations in warning signals, it is thus necessary to include both the colour and configuration of patches. We thus combined the Colour Pattern Modelling (CPM) methodology [40] that enables a precise quantification of patches' size, shape and position variation, to the perceived variation of each colour constituting the pattern. Colours were characterized taking into account humans' and birds' perception of actual butterflies' colours. This allowed us to directly test the effects of predation on warning signal polymorphism in the wild, and to accurately characterize the fitness landscape associated with polymorphic mimicry in wild populations. We address two main questions: (i) does natural selection act against intermediate phenotypes and favour genetic architecture that prevents the expression of intermediate morphs? (ii) How similar must an intermediate morph be to a mimetic homozygote to gain protection?

## 2. Material and methods

### (a) Study sites

**Figure 1.**
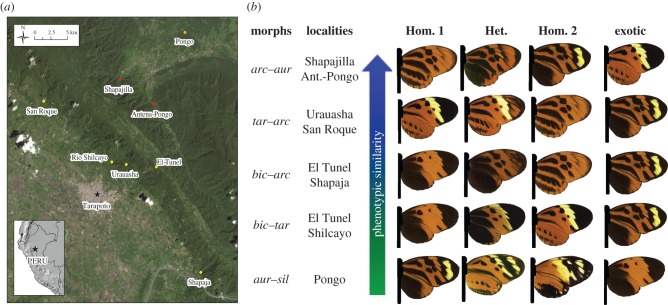
(*a*) The study area in northern Peru. Localities in yellow (San Roque, Shilcayo, Urauasha, El Tunel and Shapaja) are in the Andean cordillera, the orange locality (Pongo) is in the Amazon basin and the red localities (Shapajilla and Antena-Pongo) fall in the transition zone between the two areas. (*b*) Morphs used in the study and names of the localities where they were placed. The sets of morphs are sorted according to the phenotypic distance calculated under UV-vision between the heterozygote (Het.) and the local dominant morph (Hom. 1), from more similar (blue side of the arrow) to less similar (green side of the arrow).

Artificial butterflies were placed at eight sites in northern Peru (San Martin department; figure 1*a*), which were chosen to represent the colour pattern diversity of *H. numata* in the region. In Andean valleys (yellow points on figure 1*a*) the morphs *tarapotensis* (*tar*)*, bicoloratus* (*bic*) and *arcuella* (*arc*) are most commonly encountered, whereas in the adjacent Amazon lowlands (orange point on figure 1*a*) the morphs *aurora* (*aur*) and *silvana* (*sil*) predominate. The boundary between the Andean cordillera and lowlands forms a transitional zone characterized by a mixture of lowland and upland morphs (red points in figure 1*a*). At each site, we estimated predation on two of the local homozygote morphs, which were assumed to stand on adaptive peaks, and the correspondent heterozygote intermediate morph (figure 1*b*). Those intermediates bore different levels of resemblance to their corresponding dominant homozygote. To make wings for artificial butterflies, we selected homozygote phenotypes and intermediate, heterozygote phenotypes obtained from controlled crosses performed between *H. numata* morphs collected in the study area. The heterozygote with the most intermediate appearance was chosen for each combination. Wings from the chosen genotypes were then photographed and printed (see below).

### (b) Quantification of phenotypic distances

We used the CPM method described by Le Poul *et al.* [40] to precisely estimate phenotypic distances between each heterozygote phenotype and its corresponding dominant homozygote phenotype, as well as between each pair of local homozygotes. In CPM, each pixel of a butterfly wing image is categorized as black, orange, yellow or white. Hind and forewings are then aligned separately to an average wing colour pattern model. After alignment, the position of each pixel in the wing image is considered homologous among all individuals. The phenotypic variation is then described by principal component analysis (PCA) using binary values for the presence/absence of each colour as values for each pixel of the wing image, thus giving the same importance to each colour change (‘binary PCA’). We estimated the phenotypic similarity between homozygotes and between heterozygote and the dominant homozygote as the Euclidean distance between them, calculated using the first eight components of the PCA, which explained 99.99% of the variation. To take into account variations in the conspicuousness of the different colours, we also performed ‘perceptual’ PCAs (‘QC PCAs’), using variables representing the sensitivity of colour receptors of potential predators instead of the binary variable. Specifically, these variables were the relative amount of light captured by each photoreceptor when observing a given colour (i.e. quantum catch [43]), estimated for visual predators such as birds, under their two main types of vision: UV-vision [44] with sensitivity ranging from 300 to 700 nm and V-vision [45] with sensitivity ranging from 400 to 700 nm. Because the classification of the different mimicry rings and the initial judgement of perfect and imperfect mimicry interactions within them is performed by humans, our colour perception was also included [46]. To obtain the quantum catches of each of these observers, we applied the method described in Vorobyev & Osorio [47], assuming a Weber fraction of 0.05 for both bird vision systems and humans, their correspondent cone photoreceptor ratios (UVS = 1, SWS = 1.92, MWS = 2.68 and LWS = 2.70 for blue tit [44]; UVS = 1, SWS = 1.9, MWS = 2.2 and LWS = 2.1 for peafowl [45] and SWS = 1, MWS = 16 and LWS = 32 for humans [46]) and using the reflectance spectra measured on black, orange and yellow patches of actual wings of *H. numata tarapotensis* morph with an AvaSpec-3468 spectrophotometer (Avantes, Apeldoorn, The Netherlands) and a deuterium–halogen light source (DH-2000, Avantes) connected to a 1.5-mm-diameter sensor (FCR-7UV200-2-1.5 × 100, Avantes) inserted in a miniature black chamber. These calculations were performed with the software AVICOL [48] assuming ‘small gap’ light condition of the tropical forest of French Guiana [49] fitting the natural condition encountered by *H. numata*.

### (c) Artificial butterflies

Artificial butterflies consisted of paper wings (matte photographic paper Epson C13S041569, printed on an Epson L110 printer) attached to a malleable black wax body with a wire to fix the butterfly to vegetation, and resembled real butterflies basking with open wings. Wings were printed on both dorsal and ventral sides. Pictures of real butterfly wings were taken under standard white light conditions. The resemblance between the printed and actual wing colours was tested and maximized by comparing the reflectance of colours on the butterflies and on paper prints obtained under several paper qualities and printing settings. As described in *Quantification of phenotypic distances*, we performed ventral and dorsal measurements of the three main colours (orange, black and yellow) found in wing patterns of *H. numata* butterflies, using one specimen per morph (11 specimens). We computed the perceived contrast between actual and artificial butterflies following Vorobyev & Osorio [47], and using the software AVICOL [48] and parameters described above. Contrasts between actual and paper wings were generally below one just noticeable difference (JND), ensuring a close resemblance to actual wing colours (see the electronic supplementary material, table S1 for more detail).

### (d) Experimental design

Experiments were carried out in June and October 2013, and January, June and August 2014. At each study site, we placed groups of five artificial butterflies (figure 1*b*) at intervals of 50 m or more along a forest trail. Each group consisted of two morphs of *H. numata* frequently encountered in the local population: a dominant homozygote (1;1) and recessive homozygote (2;2), as well as a heterozygote (1;2) carrying one allele from each of the homozygotes. We also included a morph of *H. numata* not present in the local population (referred to as the ‘exotic’ morph), which was assumed to be unknown to the local predators and expected to suffer a high predation rate. Finally, a palatable cryptic butterfly (*Pierella hyceta*) of similar size to *H. numata* was included as a control, to estimate variation in predation pressure between localities. Whenever possible, we placed groups in light gaps because *Heliconius* butterflies commonly exploit those habitats. The distance between each butterfly in a group was never less than 3 m. A 4% permethrine solution was sprayed over the dummies to deter attacks by insects and arthropods. Between 180 and 300 groups were placed at each study site and collected after 72 h.

### (e) Estimation of predation

After collecting the artificial butterflies, we checked their wax bodies for marks. Birds such as jacamars and flycatchers [50], as well as other insectivorous animals such as diurnal mammals [51] and lizards [52] are known to be important visual predators of *Heliconius* butterflies. Therefore, only large marks which could have been produced by a beak (U or V shape) or by vertebrate jaws were considered as attacks. Smaller marks that were more likely left by insect mandibles were not recorded as valid attacks. Artificial butterflies that could not be found after the 72 h were excluded from the analyses. To test whether the predation intensity varied between the study sites, we compared the number of attacks registered on the palatable control across sites using a *χ*^2^-test. To test whether the phenotypes were subject to different rates of attack, we applied a generalized linear model (GLM) with all heterozygotes lumped together, with attack rate as the response, binomial error distribution, and phenotype as the predictor. The effect of the predictors was tested using ANOVA. We also tested for differences in the attack rates between phenotypes for each specific set applying a GLM. Sites at which the same morphs were tested were combined (figure 1*b*).

To estimate the phenotypic proximity necessary to benefit from mimicry protection, we used linear regression to test whether phenotypic distance (independently computed by binary PCA and by QC (perceptual) PCA for the three different observers) predicts the ratio of attacks on the heterozygote to the most similar homozygote (with the dominant alleles 1;1). We also tested the adaptive effect of peak proximity (i.e. similarity between homozygotes) on generalization by predators by regressing the heterozygote attack rate against the phenotypic distances between local homozygotes, controlling by distance to the peak. Finally, we tested whether peak proximity had an effect on peak slope, fitting and comparing GLMs having attack rate as a response variable and phenotypic distance to peak (het-homD), phenotypic distance to the other peak (het-homR, as a proxy of distance between peaks) and its interaction as predictors. If the interaction is significant, it suggests that peak proximity affects the peak slope. All the statistical analyses were performed using R [53].

## 3. Results

### (a) Identification of local fitness peaks

**Figure 2.**
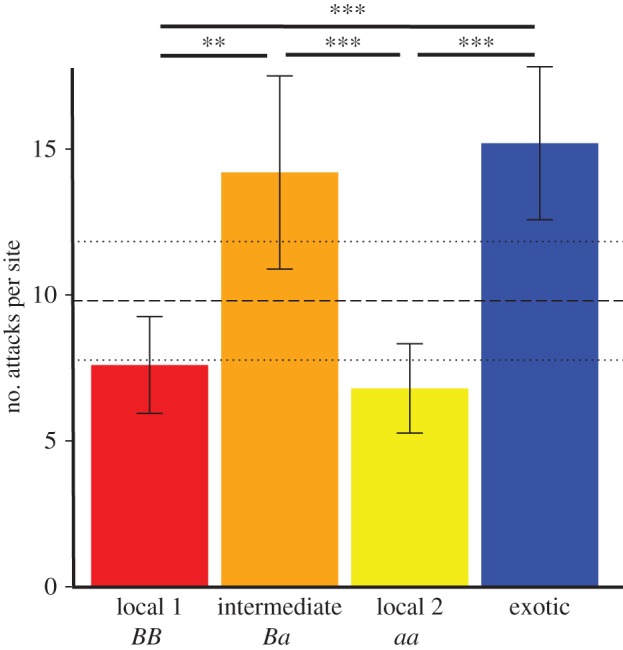
Mean and standard error of the number of attacks per site registered for the two local homozygous phenotypes, the heterozygous phenotype and the exotic phenotype. Bars with asterisks at the top refer to significant comparisons in the number of attacks between *H. numata* forms. The dashed line represents the media of attacks registered on the palatable models, and the dotted lines represent its standard error (**p < 0.01, ***p < 0.001).

Out of 5360 released dummy butterflies, 98.4% were recovered and 268 attacks were registered (i.e. 5.34% of the models displayed a clear attack mark). Predation intensity (measured as predation rate on the palatable control (*P. hyceta*)) was similar in all the localities (*χ*^2^ = 3.65, d.f. = 4, *p* = 0.46). When analysing the overall attack rates on heterozygote, homozygote, exotic and palatable artificial butterflies across all sites, the palatable control was significantly less attacked than the exotic morph (Coeff. Estim. = −0.52, s.e. = 0.19, *z* = −2.76, *p* = 0.006; figure 2), but similarly attacked to both local homozygotes (Local 1: Coef. Estim = −0.24, s.e. = 0.22, *z* = −1.09, 0.27; Local 2: Coef. Estim = −0.35, s.e. = 0.23, *z* = −1.54, *p* = 0.12). The palatable control displayed a cryptic colour pattern, and therefore benefited from a similarly reduced predation risk to local aposematic prey. Exotic morphs suffered about twice as many attacks as the local aposematic morphs (homozygote 1: Coeff. Estim. = −0.83, s.e. = 0.21, *z* = −3.98, *p* < 0.0001 and Coeff. Estim. = −0.82, s.e. = 0.21, homozygote 2: *z* = −3.94, *p* < 0.0001; figure 2). This finding confirms the ability of visual predators to discriminate known local morphs from unknown exotic morphs. Moreover, it shows that locally frequent homozygous morphs benefit from protection due to mimicry and therefore represent fitness peaks in the local fitness landscape associated with wing colour pattern phenotype.

### (b) Detecting the existence of fitness valleys

As shown in figure 2, overall, heterozygote morphs suffered on average 14.2 attacks per site (s.d. = 7.4, s.e. = 3.31). They were therefore significantly more attacked than local morphs (homozygote 1: Coeff. Estim. = −0.72, s.e. = 0.21, *z* = −3.45, *p* = 0.0006; homozygote 2: Coeff. Estim. = −0.71, s.e. = 0.21, *z* = −3.40, *p* = 0.0007) and more than the palatable butterflies (Coeff. Estim. = −0.42, s.e. = 0.19, *z* = −2.20, *p* = 0.03). Furthermore, no difference was detected between the attacks on heterozygotes and on exotic morphs (Coeff. Estim. = 0.10, s.e. = 0.17, *z* = 0.60; *p* = 0.55), suggesting that intermediate morphs were generally not recognized as known prey and were subjected to significant counter selection in natural populations. This constitutes a direct demonstration that intermediate morphs fall into fitness valleys between the adaptive peaks on which the locally common homozygous morphs stand.

### (c) Proximity to fitness peaks

**Figure 3.**
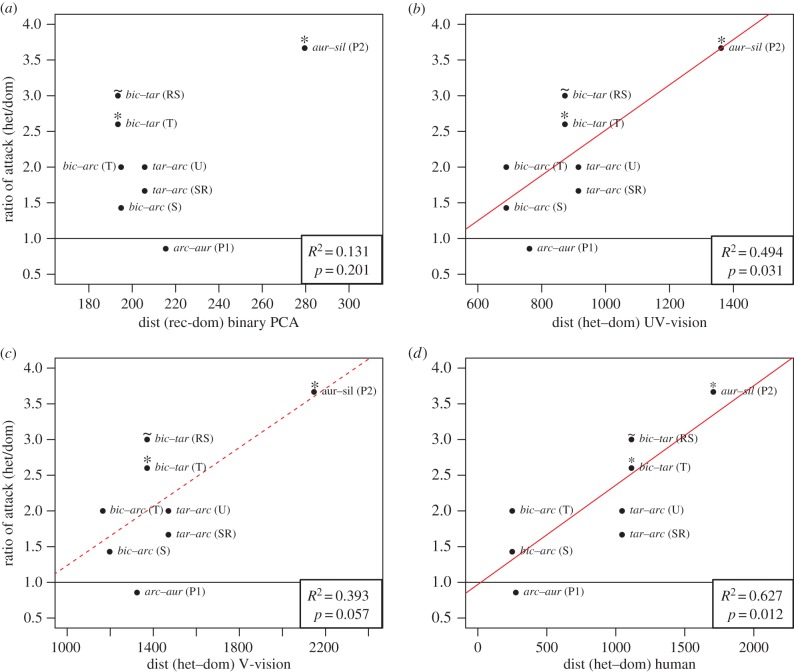
Ratio of attacks between heterozygote and dominant homozygote versus the phenotypic distances between them. When values are close to 1 (where the solid horizontal line is) the heterozygote was protected by the homozygote's signal (i.e. they had similar attack rates). For three of the five tested heterozygotes, two localities were tested (the three pairs of points that present the same *x*-value). In parenthesis are the names of the localities used (T, El Tunel; S, Shapaja; RS, Rio Shilcayo; U, Urauasha; SR, San Roque; P1, transition zone; P2, Pongo). Phenotypic distances were calculated (*a*) by binary PCA and by QC PCA including the quantum catch of (*b*) a UV-vision system (blue tit), (*c*) a V-vision system (peafowl) and (*d*) humans. A positive correlation is represented by a regression line, dashed if marginally significant and solid when significant at 0.05 level. A star over the dot (*) stands for a significant difference with *p <* 0.05 and a tilde (∼) for *p* < 0.1 in the independent linear regressions calculated for each heterozygote.

To test whether some heterozygotes could still benefit from protection conferred by imperfect resemblance to the closest local warning signal, we analysed the data for each heterozygote independently. Two out of the five tested heterozygotes were significantly more attacked than the most similar local homozygotes (electronic supplementary material, table S2). For two other tested heterozygotes, a similar trend was observed, although it was not significant (electronic supplementary material, table S2). For the remaining heterozygote tested, such a trend was not detected, probably because this set was tested in the transition zone between the Amazonian and Andean morphs (electronic supplementary material, table S2). To investigate whether the protection gained by some heterozygotes was due to greater resemblance to a local homozygote, we calculated the ratio of attacks on each heterozygote to attacks on the most similar homozygote, and then regressed this against their phenotypic similarity (figure 3). When phenotypic distance was computed using pattern variations alone (binary PCA), no correlation between the ratio of attacks and phenotypic resemblance was detected (*R*^2^ = 0.13, *F* = 2.06, *p* = 0.2; figure 3*a*). However, when phenotypic distance was computed using both pattern variations and colour conspicuousness (QC PCA), the phenotypic distance to dominant homozygotes was positively correlated with attack ratio (figure 3*b*–*d*). This correlation was significant for human observers (*R*^2^ = 0.63, *F* = 12.77, *p* = 0.012) and for birds having a UV-type vision (*R*^2^ = 0.49, *F* = 7.84, *p* = 0.03), and was marginally significant for birds having a V-type vision (*R*^2^ = 0.39, *F* = 5.53, *p* = 0.06). These results indicate that the more similar heterozygotes are to the dominant morph present in the population, the more protected they are due to generalization from predators.

### (d) Detection of fitness ridges

The rate at which heterozygotes protection increases with proximity to an adaptive peak (i.e. the slope of the peak) may depend on the presence of other peaks nearby in the adaptive landscape. Close peaks could be perceived as similar warning signals by predators, resulting in relaxed selection on the intermediate morphs falling between them and leading to shallower valleys and even ridges. The attack ratio between heterozygotes (1;2) and dominant homozygotes (1;1) was indeed correlated to the phenotypic distance between the two homozygotes (1;1) and (2;2) irrespective of which method was used to estimate phenotypic distances (binary PCA: *R*^2^ = 0.642, *F* = 13.55, *p =* 0.01; QC PCA assuming UV-type bird vision: *R*^2^ = 0.578, *F* = 10.59, *p =* 0.02; QC PCA assuming V-type bird vision: *R*^2^ = 0.634, *F* = 13.12, *p =* 0.01; QC PCA assuming human vision: *R*^2^ = 0.315, *F* = 4.22, *p =* 0.09, see the electronic supplementary material, S3 and S4 for more details). This suggests that the more similar the two local homozygotes are, the more they are generalized by predators, and the more intermediate morphs between them benefit from protection. Furthermore, it suggests the existence of a fitness ridge between close adaptive peaks (i.e. peaks separated by a short phenotypic distance). To check for the effect of resemblance of heterozygotes to the next peak and level of resemblance between peaks, we tested the effect of these two features on the attack ratio. Models including both distances and their interaction provided the best fit (lowest AIC values in electronic supplementary material, table S5). However, the interaction was significant under V-vision, but only marginally significant under UV-vision (electronic supplementary material, table S5). Both distances, but specially proximity to closest peak seemed to have an effect on intermediate phenotype protection.

## 4. Discussion

### (a) Fitness landscape associated with complex traits: the importance of colour perception

The topography of the fitness landscape associated with polymorphic mimicry depends on whether colour and/or pattern are perceived by predators as important warning signals. Behavioural experiments with birds have shown that colour plays an important role in learning and generalization by predators [17,54]. Here, we found that including information on predators' colour perception led to different fitness landscapes than those inferred using pattern variation alone, and provided a better correlation between phenotypic distance and protection. This suggests that colour changes may have a strong impact on the generalization processes exerted by birds on colour pattern variations. A striking illustration of the importance of colour can be seen in the heterozygote *bic*–*tar,* which differs from the dominant homozygote *bic–bic* by the presence of a yellow band covering a limited area of the forewing (see morphs in figure 1*b*). This variation results in a small estimated phenotypic distance when considering pattern variation alone (binary PCA), because only a small proportion of the wing varies between the two morphs. However, the estimated phenotypic distance was higher when considering colour conspicuousness (QC PCA), because the presence of a yellow band contrasting strikingly with black might be perceived by birds as an important difference [21]. Our study therefore stresses the need to quantify phenotypes as closely as possible to the way they are perceived by putative selective agents. The use of more relevant variables (such as colour perception) permits a better estimation of the fitness landscape shape, for example, by allowing the detection of continuums that can otherwise be misinterpreted as independent peaks [9]. Furthermore, our results show that the perception of phenotypic variation can differ between predators and thus modify the topography of the fitness landscape. Predator communities are probably complex [55,56], and spatially [57] and temporally variable [58]. The study of the perception of phenotypic variation by the different predators can therefore help us to understand how selection might be relaxed in certain phenotypic dimensions.

### (b) The genetic architecture of traits in fitness landscapes with multiple peaks

By quantifying the selection imposed by predators on morphs of *H. numata* butterflies in natural conditions, we provide direct evidence that selection maintains several distinct adaptive peaks within populations of the same species. The height of these adaptive peaks is related to the frequency of the different warning signals within each locality [59]. However, since we aimed at investigating how generalization capacities shape the fitness landscape, we focused on the phenotypic distance to the most similar morph rather than the most abundant. Overall, heterozygote morphs were significantly more attacked than the local homozygotes, and similarly attacked to the exotic morphs. Therefore, most intermediate morphs do not appear to benefit from protection conferred by predator generalization to the local morphs, especially when the phenotypic distance to their closest local homozygote was large. Such selection against intermediate heterozygotes fits the expectations described in Mallet's interpretation of the shifting balance theory [5,60], which states that once different selective peaks are established, intermediate phenotypes are strongly counter-selected, thus creating deep fitness valleys and maintaining several distinct peaks.

The shape of the fitness landscape may have influenced the supergene architecture underlying wing colour pattern variation in *H. numata*, which contrasts with the multilocus architecture typically observed in locally monomorphic *Heliconius* species [32]. The high predation suffered by intermediate morphs suggests selection against recombination among supergene haplotypes and co-dominance between haplotypes. Chromosomal inversions observed at the supergene [34] may therefore have been promoted, because they limit recombination. Similarly, the strict dominance relationships observed between sympatric supergene haplotypes [40] probably results from natural selection exerted against intermediates. Direct evidence of natural selection acting on genetic dominance is scarce [61], but shows that dominance may evolve in traits exhibiting adaptive polymorphism [62]. The appropriate characterization of the fitness landscapes associated with polymorphic adaptive traits thus allows a better understanding of how natural selection can shape the underlying genetic architecture.

### (c) New insights on the origin of adaptive variants inferred from fitness landscapes

The deep fitness valleys separating adaptive peaks are generally described as a barrier to the emergence of new adaptive variants [63]. However, here we found that intermediate morphs might benefit from increased protection when they reach sufficient similarity to the phenotype standing on the closest adaptive peak, thus indicating that the slopes around peaks are moderate. This suggests that once a valley has been bridged, for instance when a mutation with a large effect has occurred, mutations with small effects that confer slightly improved resemblance can be positively selected. As such, our findings present an example of how gradual and directional transitions promoted by natural selection can lead to attainment of a new adaptive peak [64]. We also demonstrated that the attack rate on intermediate morphs is positively related to phenotypic distance between local morphs, consistent with shallower fitness valleys between close adaptive peaks. The existence of fitness ridges and moderate slopes surrounding fitness peaks exemplifies how neutral variability can occur within populations without being strongly penalized by natural selection. Drift between close fitness peaks is then possible, allowing exploration of the fitness landscape and potentially the evolution of new adaptive variants, as proposed by Mallet [60]. Generalization by predators may result in relaxed selection, enabling new variants to emerge. However, the extent to which proximity between fitness peaks determines their slopes remains unclear. Although phenotypic similarity to the closest peak seems to be most important predictor of selection on intermediate phenotypes, a different experimental set-up would be required to truly disentangle the effects of peak proximity and distance between peaks.

## 5. Conclusion

By estimating selection in natural populations of *H. numata,* we characterized precisely the fitness landscape associated with polymorphic mimicry. We demonstrated the importance of colour in the evolution of colour pattern variation, thus shedding light on the importance of the different visual elements constituting this complex trait. This emphasizes how an accurate characterization of a fitness landscape can only be achieved when complex phenotypes are described using ecologically relevant dimensions. Within *H. numata*, we demonstrated the existence of adaptive peaks associated with locally abundant mimetic morphs, and surrounded by non-adaptive valleys. We show that natural selection is likely to promote genetic architecture preventing the expression of intermediate phenotypes. Examples of such architecture are completeness of genetic dominance and the absence of recombination among adaptive alleles, as is observed in *H. numata*. We also show that close fitness peaks are separated by ridges, favouring colour pattern switches and allowing drift from local peaks. Once an intermediate phenotype reaches the proximity of a peak, Fisherian transitions will facilitate climbing that peak. Further studies are required to quantify in more detail the ‘tolerance’ of the fitness landscape towards imperfect mimics that fall between distant adaptive peaks (i.e. local phenotypes with very distinct appearance).

## Supplementary Material

Supplementary information

## Data accessibility

Available on Dryad http://dx.doi.org/10.5061/dryad.g8723.
