# Supplementary material for: Crossing fitness valleys: empirical estimation of a fitness landscape associated with polymorphic mimicry
Source: Proc Biol Sci. 2016 Apr 27;283(1829):20160391. doi: 10.1098/rspb.2016.0391 (PMC4855388; doi:10.1098/rspb.2016.0391)
Supplement: Supplementary information [file rspb20160391supp1.docx]

**Supplementary information**

**S1.** Chromatic (DS) and achromatic (DQ) differences in orange, black and yellow between the real wing colours and the printed colours. Differences were computed under the two main visual systems known in birds (UV-type: Blue Tit and V-type: Peafowl) and using a “small gap” type of light. We chose the printing settings that allowed us to have colour combinations as similar as possible to the real ones (Delta S and Delta Q less or not much higher than 1).

|  |  | UV-vision | | V-vision | |
| --- | --- | --- | --- | --- | --- |
|  |  | DS | DQ | DS | DQ |
| Int. BIC-TAR | black | 0.27 | 0.24 | 0.31 | 0.34 |
|  | orange | 0.46 | 0.02 | 0.78 | 0.06 |
|  | yellow | 1.19 | 0.77 | 1.03 | 1.65 |
| Int. AUR-ARC | black | 0.28 | 0.26 | 0.33 | 0.37 |
|  | orange | 0.38 | 0.01 | 0.63 | 0.00 |
| Int. TAR-ARC | black | 0.65 | 0.08 | 0.53 | 0.14 |
|  | orange | 0.77 | 0.15 | 0.96 | 0.20 |
|  | yellow | 1.47 | 0.34 | 0.90 | 0.65 |
| Int. SIL-AUR | black | 0.25 | 0.11 | 0.28 | 0.14 |
|  | orange | 0.20 | 0.11 | 0.48 | 0.15 |
|  | yellow | 1.41 | 0.20 | 1.17 | 0.37 |
| Int. BIC-ARC | black | 0.28 | 0.25 | 0.32 | 0.36 |
|  | orange | 0.42 | 0.02 | 0.71 | 0.03 |

**S2.** Results of generalised linear models comparing attack rates on heterozygotes to the local and exotic morphs (Dom for dominant and Rec for recessive) for each of the tested combinations. ** *p* < 0.01, * *p* < 0.05, ~ *p* < 0.1.

|  |  |  | | Heterozygote | | | | |  |  | | Exotic | | | | |
| --- | --- | --- | --- | --- | --- | --- | --- | --- | --- | --- | --- | --- | --- | --- | --- | --- |
| Tested het. | Morph | | Estimate | | Std. Error | *z* | *p* | | Morph | | Estimate | | Std. Error | *z* | *p* | |
| *sil-aur* | Dom | | -1.37 | | 0.66 | -2.06 | 0.04 | * | Dom | | -1.36 | | 0.66 | -2.05 | 0.04 | * |
|  | Exotic | | -0.01 | | 0.44 | -0.02 | 0.99 | NS | Het | | 0.01 | | 0.44 | 0.02 | 0.99 | NS |
|  | Rec | | -0.64 | | 0.52 | -1.50 | 0.13 | NS | Rec | | -0.63 | | 0.52 | -1.49 | 0.14 | NS |
| *bic-tar* | Dom | | -1.11 | | 0.40 | -2.75 | 0.006 | *** | Dom | | -0.93 | | 0.41 | -2.26 | 0.02 | * |
|  | Exotic | | -0.17 | | 0.31 | -0.55 | 0.58 | NS | Het | | 0.17 | | 0.31 | 0.55 | 0.58 | NS |
|  | Rec | | -0.12 | | 0.40 | -2.77 | 0.01 | ** | Rec | | -0.94 | | 0.41 | -2.29 | 0.02 | * |
| *arc-aur* | Dom | | 0.17 | | 0.57 | 0.24 | 0.77 | NS | Dom | | -0.81 | | 0.47 | -1.71 | 0.09 | ~ |
|  | Exotic | | 0.97 | | 0.50 | 1.96 | 0.05 | ~ | Het | | -0.97 | | 0.50 | -1.96 | 0.05 | ~ |
|  | Rec | | 0.02 | | 0.59 | 0.04 | 0.97 | NS | Rec | | -0.95 | | 0.50 | -1.91 | 0.06 | ~ |
| *arc-tar* | Dom | | -0.65 | | 0.52 | -1.26 | 0.21 | NS | Dom | | -0.33 | | 0.55 | -0.59 | 0.55 | NS |
|  | Exotic | | -0.33 | | 0.47 | -0.69 | 0.49 | NS | Het | | 0.34 | | 0.47 | 0.69 | 0.49 | NS |
|  | Rec | | -1.75 | | 0.77 | -2.25 | 0.02 | * | Rec | | -1.42 | | 0.80 | -1.78 | 0.08 | ~ |
| *bic-arc* | Dom | | -0.52 | | 0.39 | -0.94 | 0.35 | NS | Dom | | -0.78 | | 0.38 | -2.04 | 0.04 | * |
|  | Exotic | | 0.25 | | 0.33 | 0.77 | 0.44 | NS | Het | | -0.25 | | 0.33 | -0.77 | 0.44 | NS |
|  | Rec | | -0.35 | | 0.37 | -1.35 | 0.18 | NS | Rec | | -0.60 | | 0.36 | -1.65 | 0.10 | ~ |

**S3**. Correlations between the ratio of attacks on heterozygote to dominant homozygote and 1) the phenotypic distance between them (het-homD); and 2) the phenotypic distance between the dominant and the recessive homozygotes (homD-homR). The phenotypical distances were calculated using binary PCA and QC PCA considering the colour perception of UV-vision (blue tit), V-vision (peafowl) and human. ** *p* < 0.01, * *p* < 0.05 and ~ *p* < 0.1.

|  | Bin. PCA | UV-vision | V-vision | Human |
| --- | --- | --- | --- | --- |
| het-homD | *R^2^*= 0.13  *p* = 0.20 | *R^2^*= 0.49  *p* = 0.03* | *R^2^*= 0.39  *p* = 0.06~ | *R^2^*= 0.63  *p* = 0.01** |
| homD-homR | *R^2^*= 0.64  *p* = 0.01** | *R^2^*= 0.58  *p* = 0.02* | *R^2^*= 0.63  *p* =0.01** | *R^2^*= 0.31  *p* = 0.09~ |

a.

b.

c.

d.

*

~

*

*

~

*

~

*

~

*

*

*

*R^2^*=0.578

*p*=0.017

*R^2^*=0.642

*p*=0.010

*R^2^*=0.315

*p*=0.086

*R^2^*=0.634

*p*=0.011

**S4**. Ratio of attack between heterozygote and dominant homozygote vs. the phenotypic distances between local homozygotes. When the values are close to 1 (where the solid line is) the heterozygote was protected by the dominant homozygote’s signal (similar attack rates). For three of the five tested sets, two localities were tested (the three couple of points that present the same x-value). In parenthesis are the names of the used localities (T: El Tunel, S: Shapaja, RS: Rio Shilcayo, U: Urauasha, SR: San Roque, P1: transition zone, P2: Pongo). Distances were calculated (**a**) by binary PCA and by QC PCA including the quantum catch of (**b**) a UV-vision system (blue tit), (**c**) a V-vision system (peafowl) and (**d**) humans. A positive correlation is represented by the red line, dashed if it is marginally non-significant and solid when it is significant at 0.05 level. A star over the dot (*) stands for a significant difference with *p <* 0.05 and a tilde (~) for *p* < 0.1, from the independent linear regressions calculated for each heterozygote.

**S5.** Comparison of GLMs explaining ratio of attack between heterozygote and dominant phenotypes. Model A included phenotypic distance to dominant (het-homD) + phenotypic distance to recessive (het-homR) + interaction between phenotypic distances. Model B included only the phenotypic distances without their interaction. Model C included only phenotypic distance between heterozygote and dominant homozygote (het-homD). Model D included only phenotypic distance between heterozygote and recessive homozygote (het-homR). The models were fitted for the phenotypic distances calculated with the binary PCA, and with the QC PCA considering UV-vision, V-vision and human’s vision. *** *p* < 0.001, ** *p* < 0.01, ~ *p* < 0.1.

|  | Bin. PCA | | | | | | | UV-vision | | | | | V-vision | | | | | Human | | | | |
| --- | --- | --- | --- | --- | --- | --- | --- | --- | --- | --- | --- | --- | --- | --- | --- | --- | --- | --- | --- | --- | --- | --- |
|  | Res  df | AIC | *Sum*  *Sq* | | | *p* |  | Res df | AIC | *Sum Sq* | *p* |  | Res df | AIC | *Sum Sq* | *p* |  | Res  df | AIC | *Sum*  *Sq* | *p* |  |
| Model A | 1 | 1.18 |  |  | | |  | 1 | 8.43 |  |  |  | 1 | -22.03 |  |  |  | 1 | 8.69 |  |  |  |
| Model B | 2 | 1.95 | -0.04 | | 0.39 | |  | 2 | 14.24 | -0.80 | 0.052 | ~ | 2 | 8.69 | -0.33 | <0.001 | *** | 2 | 10.23 | -0.23 | 0.31 |  |
| Model C | 3 | 17.43 | -2.80 | | <0.001 | | *** | 3 | 13.63 | 0.33 | 0.42 |  | 3 | 14.89 | 1.40 | 0.004 | ** | 3 | 10.98 | 0.33 | 0.23 |  |
| Model D | 3 | 7.97 | -0.35 | | 0.005 | | ** | 3 | 12.69 | 0.10 | 0.66 |  | 3 | 10.73 | 0.42 | 0.11 |  | 3 | 16.48 | 1.92 | 0.004 | ** |
